# Prognostic Impact of Overhydration in Hospitalized Patients Receiving Parenteral Nutrition

**DOI:** 10.3390/nu18213528

**Published:** 2026-10-26

**Authors:** Alba Zabalegui-Eguinoa, Fernanda Mucarzel-Suarez-Arana, Raül Cartiel-Vesperinas, Cora Oliver-Vila, Marta Ricart-Viles, Laura Andurell-Ferrandiz, Amador Lluch-Romero, Albert Puig-Perez, Fiorella Palmas, Rosa Burgos-Peláez

**Affiliations:** 1 Nutrition Support Unit, Endocrinology and Nutrition Department, Hospital Universitari Vall d’Hebron, 08035 Barcelona, Spain; alba.zabalegui@vallhebron.cat (A.Z.-E.); raul.cartiel@vhir.org (R.C.-V.); marta.ricart@vallhebron.cat (M.R.-V.); laura.andurell@vallhebron.cat (L.A.-F.); amador.lluch@vallhebron.cat (A.L.-R.); fiorellaximena.palmas@vallhebron.cat (F.P.); rosa.burgos@vallhebron.cat (R.B.-P.); 2 ISS IR-HUVH-VHIR Diabetes and Metabolism Research Group, Endocrinology Department, Vall d’Hebron University Hospital, Autonomous University of Barcelona, 08035 Barcelona, Spain; cora.oliver@vhir.org (C.O.-V.); albert.puig@vhir.org (A.P.-P.); 3 Department of Pharmacology, Autonomous University of Barcelona, 08193 Bellaterra, Cerdanyola del Vallès, Barcelona, Spain

**Keywords:** parenteral nutrition, bioelectrical impedance analysis, fluid overload, in-hospital mortality, BIVA

## Abstract

**Background & Aims:** Accurate assessment of hydration status is particularly relevant in hospitalized patients receiving parenteral support in whom fluid disturbances are common and may be associated with adverse outcomes. However, the prognostic significance of bioimpedance-derived hydration status in the population receiving parenteral nutrition (PN) remains poorly defined. This study aimed to evaluate the association between hydration status at the beginning of PN and in-hospital mortality. **Methods:** This single-center prospective observational cohort included hospitalized adults receiving PN between November 2024 and March 2026. Hydration status was assessed within 72 h of PN initiation using bioelectrical impedance analysis (Hydragram^®^, Akern). Receiver operating characteristic (ROC) analysis was used to identify the optimal hydration threshold for in-hospital mortality prediction. Survival was evaluated using Kaplan–Meier analysis and Cox proportional hazards models. **Results:** A total of 276 patients were included (62.0% male; mean age 64.9 ± 17.5 years). Overall in-hospital mortality was 13.4%. Hydration percentage showed good discrimination for in-hospital mortality (AUC 0.776, 95% CI 0.672–0.880; *p* < 0.001), with an optimal cut-off value of 79.45%. Patients with overhydration exhibited higher mortality rates than normohydrated patients (29.3% vs. 5.4%; log-rank *p* < 0.001). In multivariable Cox regression analysis, overhydration remained independently associated with in-hospital mortality (HR 2.85, 95% CI 1.21–6.70; *p* = 0.016), together with age (HR 1.06, 95% CI 1.02–1.10; *p* = 0.002). Bioelectrical impedance vector analysis (BIVA) demonstrated a clustering of non-survivors in vector regions consistent with extracellular fluid expansion. **Conclusions:** Overhydration assessed by bioelectrical impedance analysis was independently associated with in-hospital mortality in patients receiving PN. Early assessment of hydration status by bioelectrical impedance may provide clinically relevant information for risk stratification in hospitalized patients receiving parenteral nutrition.

## 1. Introduction

Fluid balance disturbances are highly prevalent among hospitalized patients and represent a major challenge in clinical practice. In critically ill patients and individuals with chronic kidney disease, both fluid overload and dehydration have been associated with adverse outcomes, including impaired organ function, prolonged hospital stay, increased healthcare utilization, and higher mortality rates [1,2,3]. Accurate assessment of hydration status is therefore essential for risk stratification and clinical decision-making, particularly in patients with acute illness and complex nutritional needs [1].

Patients requiring parenteral nutrition (PN) constitute a particularly vulnerable population. These individuals frequently present with severe underlying disease, systemic inflammation, gastrointestinal dysfunction, malnutrition, and altered fluid homeostasis [4,5]. Furthermore, the initiation and management of PN often involve substantial intravenous fluid administration, which may contribute to changes in body water distribution and increase the risk of fluid imbalance [6]. Consequently, monitoring hydration status is an integral component of nutritional and metabolic care in hospitalized patients receiving PN, although no data are available currently in this population.

Despite its clinical relevance, the assessment of hydration status remains challenging. Conventional methods, including physical examination, body weight changes, fluid balance charts, and biochemical parameters, have important limitations and may fail to accurately reflect body fluid compartments [7,8]. In this context, bioelectrical impedance analysis (BIA) has emerged as a practical, non-invasive, and bedside tool capable of providing objective information on body composition and hydration status. Beyond its established role in nutritional assessment, BIA has demonstrated utility in detecting fluid overload and monitoring changes in body water distribution across a variety of clinical settings [9,10,11].

In addition to its direct clinical consequences, altered hydration status may substantially affect the interpretation of nutritional assessment parameters in hospitalized patients [12]. Individuals requiring PN frequently present with systemic inflammation and acute-phase responses, characterized by elevated C-reactive protein (CRP) concentrations and reduced levels of negative acute-phase reactants such as prealbumin [13]. Consequently, the CRP-to-prealbumin ratio has emerged as an integrated marker reflecting both inflammatory burden and nutritional status, and has been associated with adverse clinical outcomes in hospitalized patients [14,15]. However, extracellular fluid expansion may further influence the interpretation of these biomarkers through dilutional effects, while simultaneously masking weight loss and altering body composition estimates, thereby complicating the diagnosis and severity assessment of malnutrition [16].

Among the bioimpedance-derived tools used to assess hydration status, the Hydragram^®^ (Akern SRL, Florence, Italy) has gained increasing clinical interest. By expressing hydration as the ratio between total body water and fat-free mass (TBW/FFM), it provides a visual and quantitative assessment of fluid balance that may facilitate the identification of patients with fluid overload [17,18]. Given the recognized association between fluid imbalance and adverse outcomes in several clinical settings, Hydragram-derived hydration measurements may offer valuable prognostic information beyond traditional nutritional and biochemical markers. Nevertheless, their prognostic significance in hospitalized patients requiring parenteral nutrition has not been specifically investigated.

These limitations are particularly relevant when body composition is assessed by bioelectrical impedance analysis [19]. Because impedance-derived estimates of fat-free mass are strongly influenced by body water distribution, fluid overload may lead to overestimation of fat-free mass and fat-free mass index (FFMI), potentially obscuring underlying muscle depletion [20]. This issue is especially important in patients receiving PN, who often present concomitant inflammation, malnutrition, and fluid imbalance. For this reason, opportunistic computed tomography (CT)-derived body composition assessment may provide complementary information, as CT-based measures of muscle quantity and quality are less affected by alterations in hydration status. However, data exploring the interaction between hydration status, body composition assessment, and clinical outcomes in patients receiving PN remain limited.

Previous studies have shown that fluid overload assessed by BIA is associated with worse clinical outcomes in patients with chronic kidney disease, heart failure, critical illness, and cancer [12,21,22,23,24]. However, evidence regarding the prognostic significance of hydration status in hospitalized patients receiving PN remains scarce. In particular, the relationship between bioimpedance-derived hydration parameters and clinically relevant outcomes, such as length of stay and intra-hospital mortality, has not been adequately characterized in this population.

We hypothesized that impaired hydration status, as assessed by BIA, would be associated with worse clinical outcomes, particularly increased in-hospital mortality, in hospitalized patients in the first 72 h of PN support.

Therefore, the aim of the present study was to investigate the prognostic value of bioimpedance-derived hydration status in hospitalized patients receiving parenteral nutrition, with a particular focus on its association with in-hospital mortality.

## 2. Materials and Methods

### 2.1. Study Design

This was a single-center prospective observational cohort. Consecutive hospitalized patients receiving parenteral nutrition were followed by the Nutrition Support Unit as part of standard clinical care between November 2024 and March 2026 at Vall d’Hebron University Hospital (Barcelona, Spain). No formal sample size calculation was performed, as all eligible consecutive patients were included to minimize selection bias. No interventions beyond routine clinical practice were performed. The study was conducted in accordance with the Declaration of Helsinki and approved by the local Research Ethics Committee of Vall d’Hebron University Hospital (reference PR(AG)510/2021).

### 2.2. Patient Selection

**Figure 1:**
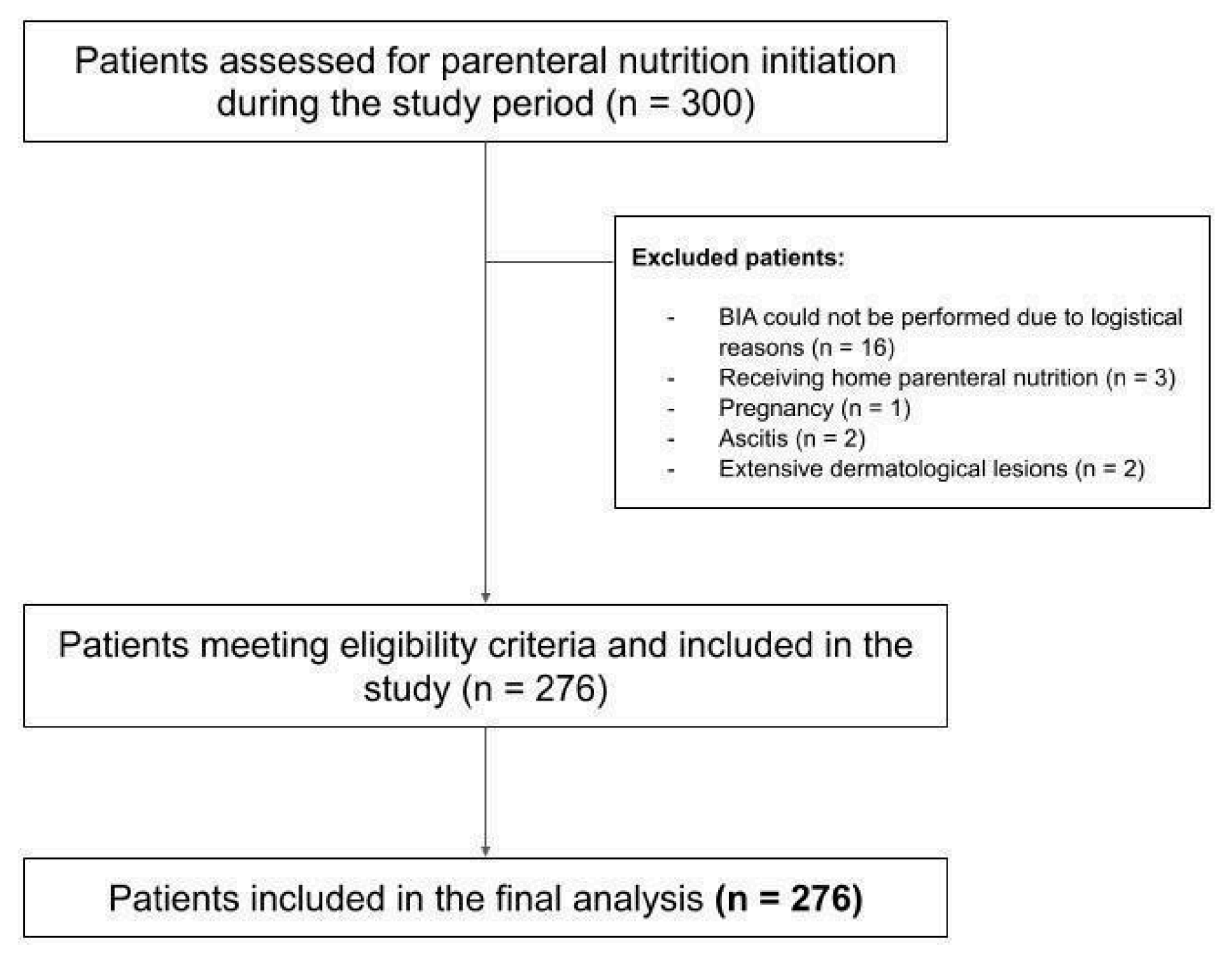
Flow-chart of patient selection process.

Consecutive adult hospitalized patients who were unable to use their gastrointestinal tract and referred to the Nutrition Unit for initiation of parenteral nutrition (PN) were considered eligible for inclusion. As part of routine clinical practice, body composition and hydration was assessed by bioelectrical impedance analysis (BIA) between 72 h after PN initiation. The patient selection process is shown in Figure 1.

When available, opportunistic abdominal Computed Tomography (CT) scans performed within a clinically relevant time interval surrounding the BIA assessment (<7 days) were used to derive additional body composition parameters. CT-derived measurements were not required for study participation and were analyzed in the corresponding subgroup of patients.

Exclusion criteria comprised patients receiving home parenteral nutrition at baseline, a hospital stay shorter than 72 h, pregnancy, extensive dermatological lesions preventing BIA assessment, significant fluid extravasation, localized hematomas affecting electrode placement, ascites, amputations and patients admitted to the intensive care unit (ICU) or managed in specialized units for heart failure or renal dysfunction who could not be assessed by our team at PN initiation. These patients were excluded because their clinical management and the associated organizational workflow precluded BIA assessment within the first 72 h after PN initiation, as required by the study protocol. Furthermore, patients in whom BIA could not be performed within the first 72 h after initiation of parenteral nutrition for logistical or organizational reasons were also excluded.

### 2.3. Clinical Data Collection

At the time of referral to the Nutrition Support Unit for parenteral nutrition (PN) initiation, all patients underwent a comprehensive clinical and nutritional assessment according to routine institutional practice. Data collected included demographic characteristics, admission category (medical or surgical), relevant comorbidities, clinical variables, and anthropometric measurements.

During the first 72 h after PN starts, bioelectrical impedance analysis (BIA) was performed to evaluate energy requirements, nutritional and hydration status under routine clinical conditions. Intravenous fluid administration previous to PN was managed according to hospital standard care (generally 1000 mL of 0.9% normal saline and from 1000 to 1500 mL of 10% dextrose solution). Besides when PN starts, the fluid administration is adjusted to 1000 mL of 0.9% normal saline plus 1000 mL of PN the first day and 1250 to 1500 mL the next 2–3 days. However, fluid intake and output data were not systematically collected.

Database construction, and statistical analyses were performed retrospectively after completion of patient follow-up.

#### 2.3.1. Nutritional and Morphofunctional Assessment

Anthropometric variables included height (m), weight (kg), Body Mass Index (BMI, kg/m^2^), and percentage of unintentional weight loss during the previous six months.

Nutritional risk was screened with Nutritional Risk Screening 2002 (NRS-2002) as the first-step screening tool [25]. Malnutrition was diagnosed according to the Global Leadership Initiative on Malnutrition (GLIM) criteria [26]. Given the acute or chronic disease-related inflammatory condition requiring hospitalization and specifically PN support, all patients fulfilled at least one etiologic criterion.

The phenotypic criterion of reduced muscle mass was assessed using the Fat-Free Mass Index (FFMI) obtained by bioelectrical impedance analysis (BIA). Low muscle mass was defined according to consensus-recommended cut-off values of FFMI < 17 kg/m^2^ for men and <15 kg/m^2^ for women [27].

Risk of refeeding syndrome was assessed according to established guideline criteria, including prolonged inadequate nutrition intake, low BMI, significant recent weight loss, and other recognized clinical factors [28].

#### 2.3.2. Bioelectrical Impedance Analysis (BIA)

Body composition was assessed by bioelectrical impedance analysis during the 72 h after parenteral nutrition started using a phase-sensitive 50 kHz impedance analyzer (Akern BIA-101/Nutrilab analyzer, Akern SRL, Pontassieve, Florence, Italy). Measurements were performed using a standardized tetrapolar electrode configuration on the right hand and foot, delivering an alternating current of 800 µA.

BIA was performed in accordance with recent standards for body composition analysis [29]. All assessments were conducted in the morning by the trained nutrition professionals with patients in the supine position. Participants remained at rest for at least 5 min before measurement in order to establish BIA values [±2 Ω for Resistance (Rz) and ±1 Ω for Xc (Reactance)], as fluid changes occurred after the change from standing to recumbency directly affected R and Z measurements.

Instrument calibration and technical accuracy were verified daily according to the manufacturer’s recommendations.

The technical accuracy of the instrument was verified daily with a precision check provided by the manufacturer.

The following bioelectrical and body composition parameters derived from Bioelectrical Impedance were recorded: Resistance (Rz, Ω), Reactance (Xc, Ω), Phase Angle (PhA, degrees), Fat-Free Mass (FFM, Kg), Fat Mass (FM, kg), Body Cell Mass (BCM), Total Body Water (TBW, %), Extra Cellular Water (ECW, %), Intra Cellular Water (ICW, %), Fat-Free Mass Index in Kg/m^2^ (FFMI), Appendicular Skeletal Muscle Mass (ASMM, kg), Sodium-to-potassium ratio (Na/k) and Basal Metabolic Rate (BMR, kcal per day).

**Figure 2:**
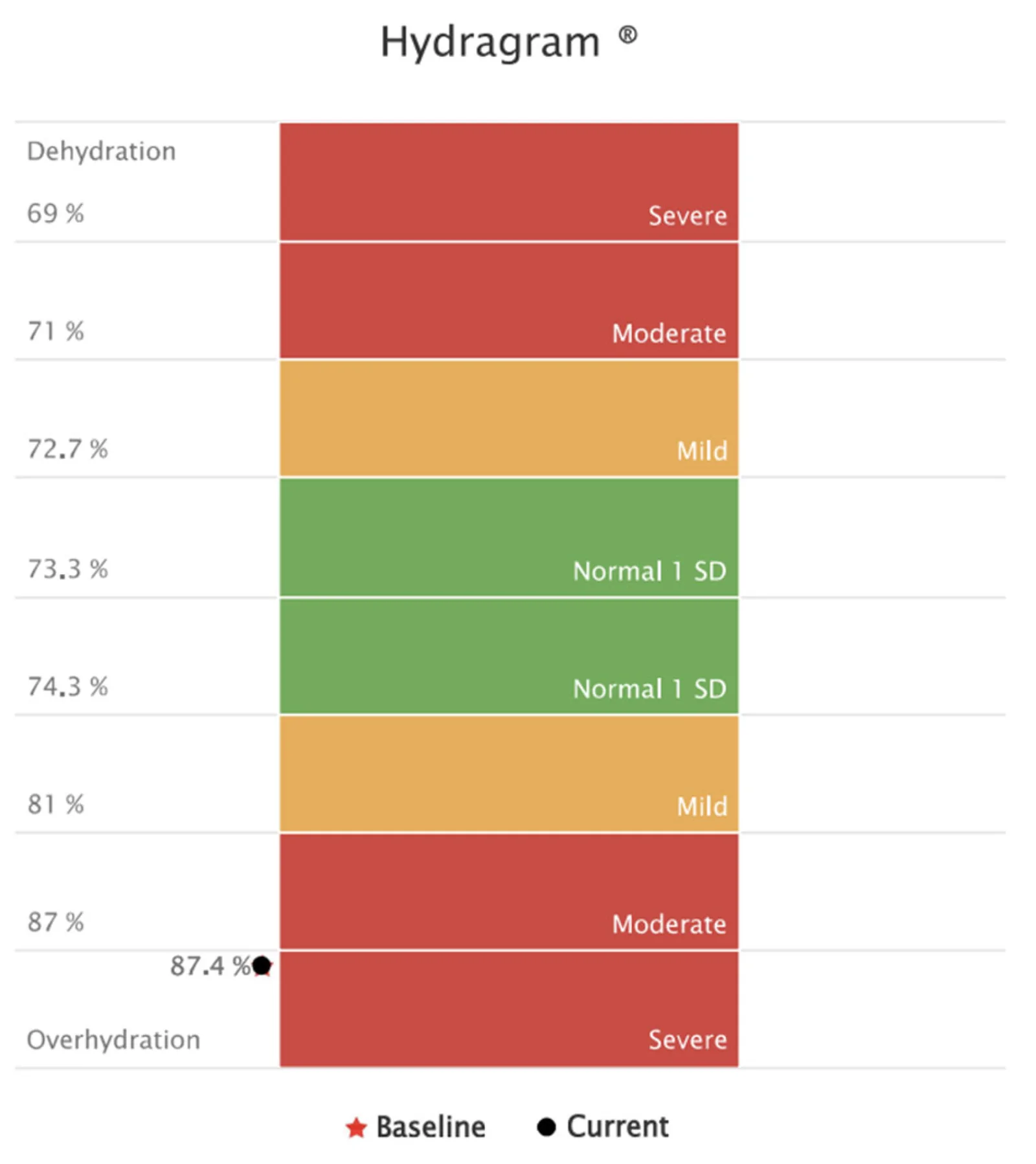
Hydragram^®^ plot used for the assessment of hydration status. The graph displays hydration percentage (TBW/FFM) relative to reference values, with displacement toward the upper region indicating relative dehydration and toward the lower region indicating fluid overload.

Hydration percentage was derived from the ratio between total body water and fat-free mass (TBW/FFM) and interpreted using the Hydragram^®^ graphical model (Akern SRL HBO, Software ver. 2.1.19), which provides a visual representation of the patient’s hydration status relative to physiological reference ranges (Figure 2) [17,30]. Values above the reference range were considered indicative of fluid overload, whereas lower values suggested relative dehydration. Hydration percentage was subsequently evaluated as a continuous prognostic marker and as a categorical variable according to the optimal threshold identified by receiver operating characteristic (ROC) curve analysis.

Phase angle was calculated as arctangent (Xc/R) × (180/π). In addition, a standardized phase angle (SPA) was calculated using age- and sex-specific reference values by subtracting the corresponding reference phase angle from the observed value and dividing the result by the reference standard deviation [30].

Bioelectrical impedance vector analysis (BIVA) was performed according to the method described by Piccoli et al. [30]. Individual impedance vectors were standardized for age and sex and plotted using dedicated BIVA software against the reference tolerance ellipses (50th, 75th, and 95th percentiles) derived from a healthy adult population. BIVA was used as a complementary tool to visually characterize the distribution of hydration and cellularity patterns according to clinical outcomes.

#### 2.3.3. Computed Tomography Derived Body Composition

When clinically indicated abdominal computed tomography (CT) scans were available as part of routine patient care, opportunistic body composition analysis was considered. No CT examinations were performed specifically for study purposes or for body composition assessment.

For patients with available CT imaging acquired within a clinically relevant time window surrounding the BIA assessments (less than 7 days), body composition was evaluated using transverse images at the level of the third lumbar vertebra (L3). Skeletal muscle and abdominal adipose tissue areas were quantified using FocusedON-BC software (Version 2.1).

**Figure 3:**
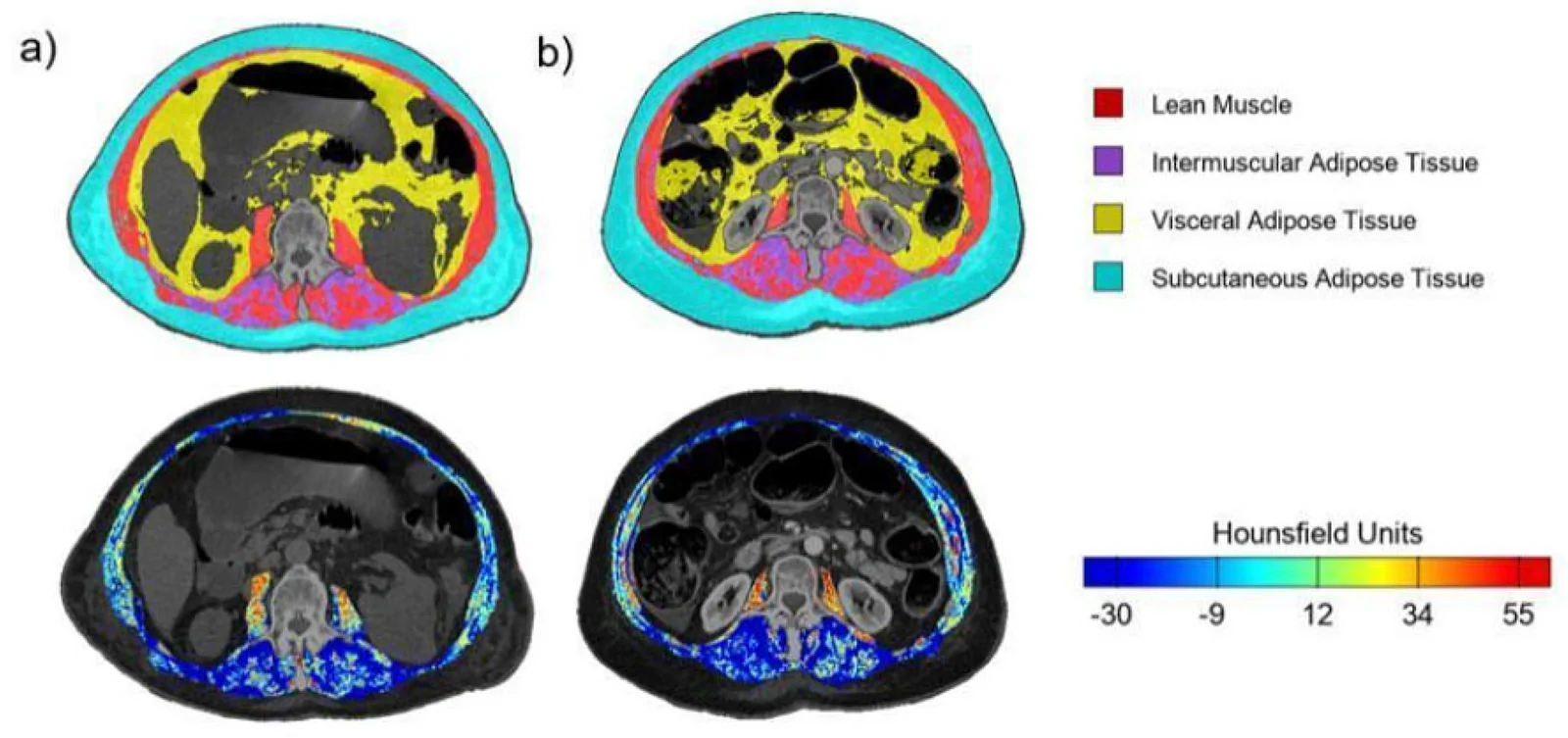
CT-derived assessment of body composition and muscle quality in a severely overhydrated male (**a**) and female (**b**) patient. Upper panels show automated tissue segmentation at the third lumbar vertebra (L3) performed using the FocusedON-BC semi-automated software, whereas lower panels display skeletal muscle radiodensity maps expressed in Hounsfield Units (HU). Despite presenting comparable severe fluid overload (88.3% hydration by bioelectrical impedance analysis), both patients exhibited markedly reduced skeletal muscle density (SMD: −19.92 HU in the male patient and −21.81 HU in the female patient), indicative of impaired muscle quality. Notably, these alterations occurred despite the preservation of skeletal muscle mass, as reflected by skeletal muscle index (SMI) values of 47.69 cm^2^/m^2^ in the male patient and 57.7 cm^2^/m^2^ in the female patient. These representative cases illustrate the dissociation between muscle quantity and muscle quality, as well as the coexistence of severe overhydration and adverse clinical outcomes.

CT images had been previously acquired for diagnostic or therapeutic indications using multidetector CT scanners, and Digital Imaging and Communications in Medicine (DICOM) files were retrieved from the Radiology Department. Tissue segmentation was performed by trained personnel using the semi-automated segmentation tool integrated within the FocusedON-BC software, allowing identification and quantification of skeletal muscle and adipose tissue compartments (Figure 3).

CT-derived body composition parameters were analyzed only in the subgroup of patients with available imaging and were not required for study inclusion.

The abdominal muscle groups analyzed included the psoas, erector spinae, quadratus lumborum, transversus abdominis, external and internal obliques, and rectus abdominis. Adipose tissue was categorized into subcutaneous, visceral, and intermuscular compartments [31,32].

The variables recorded included Skeletal Muscle Index (calculated by Skeletal Muscle Area (SMA) normalized to the patient’s height squared, SMI in cm^2^/m^2^), Lean Muscle Index (calculated by Lean Muscle Area (LMA) normalized to the patient’s height squared, LMI in cm^2^/m^2^), InterMuscular Adipose Tissue Index (calculated by InterMuscular Adipose Tissue Area (IMAT) normalized to the patient’s height squared, IMATI, in cm^2^/m^2^), Visceral Adipose Tissue Index (calculated by Visceral Adipose Tissue Area (VAT) normalized to the patient’s height squared, VATI, in cm^2^/m^2^), and, Subcutaneous Adipose Tissue Index (calculated by Subcutaneous Adipose Tissue Area (SAT) normalized to the patient’s height squared, SATI, in cm^2^/m^2^).

Tissue quality for muscle mass was assessed based on its radiodensity (D) measured by mean of Hounsfield Units (HU), applying standard radiodensity thresholds: −29 to 150 HU for skeletal muscle mass, −190 to −30 for SAT and IMAT and −150 to −50 for VAT [33].

#### 2.3.4. Outcome Measures

Clinical outcomes collected during follow-up included length of hospital stay, discharge destination, occurrence of refeeding syndrome and mortality.

Length of hospital stay was defined as the number of days from hospital admission to discharge or death. Discharge destination was categorized as home, Hospital-at-Home (HaH) program, intermediate care facility or in-hospital death.

The occurrence of refeeding syndrome was prospectively recorded during hospitalization according to institutional clinical practice and predefined diagnostic criteria [28].

Mortality outcomes included both in-hospital mortality and all-cause mortality within 6 months after hospital discharge. Vital status was determined through review of electronic medical records and follow-up clinical documentation. For patients who died during the hospitalization, the date of death was recorded as in-hospital mortality. For discharged patients, deaths occurring within 6 months after discharge were recorded as post-discharge mortality.

### 2.4. Statistical Analysis

Continuous variables were expressed as mean ± standard deviation (SD) for normally distributed data or as median and interquartile range (IQR) for non-normally distributed data. Normality was assessed using the Shapiro–Wilk test. Categorical variables were summarized as frequencies and percentages [*n* (%)].

Comparisons between groups were performed using Student’s *t*-test or Mann–Whitney U test for continuous variables, as appropriate, and the Chi-square test or Fisher’s exact test for categorical variables.

To explore the relationship between hydration status and mortality, bioelectrical impedance parameters obtained 72 h after PN initiation were compared between survivors and non-survivors. Receiver operating characteristic (ROC) curve analysis was performed to evaluate the ability of hydration percentage to predict in-hospital mortality and to identify the optimal cut-off value according to the Youden index. The area under the curve (AUC) and corresponding 95% confidence intervals (95% CI) were calculated.

Survival according to hydration status was assessed using Kaplan–Meier curves and compared using the log-rank test. Patients were classified according to the ROC-derived hydration threshold.

To identify independent predictors of mortality, multivariable Cox proportional hazards regression models were constructed including clinically relevant covariates and variables associated with mortality in univariate analyses. Results are reported as hazard ratios (HR) with 95% confidence intervals (95% CI).

Missing data were assessed prior to statistical analysis. Complete-case analysis was performed for all inferential analyses, and no data imputation was applied. Missing data were limited to opportunistic CT-derived BC variables (7.2%), reflecting the absence of clinically indicated CT examinations within the predefined time window rather than loss to follow-up. Therefore, analyses involving CT-derived variables were restricted to patients with available imaging data.

## 3. Results

### 3.1. Study Population

A total of consecutive 276 hospitalized patients requiring parenteral nutrition were included in the study, comprising 169 men (61.2%) and 107 women (38.8%). Baseline demographic, nutritional and body composition characteristics according to sex are presented in Table 1.

No significant sex-related differences were observed regarding age, BMI, admission category, prevalence of oncological disease, duration of fasting prior to PN initiation, or prevalence of GLIM-defined malnutrition. Men exhibited significantly higher FFMI values than women (18.81 ± 2.63 vs. 17.14 ± 2.52 kg/m^2^, *p* < 0.001). However, low FFMI was more prevalent among men (23.7% vs. 11.2%, *p* = 0.010) according to GLIM specific cut-off points.

Opportunistic CT analysis (n = 256) revealed sex-related differences in several body composition parameters, including skeletal muscle index, skeletal muscle radiodensity and visceral and subcutaneous adipose tissue indexes.

### 3.2. Follow-Up During the 72 h After PN Initiation

At 72 h after PN initiation, mean hydration values were 77.0 ± 5.2% in men and 78.2 ± 6.5% in women without differences by sex (*p* = 0.093).

Men showed higher phase angle values, body cell mass, nutritional index and basal metabolic rate than women, whereas women presented lower resistance values. Hydration percentage did not differ significantly between sexes (*p* = 0.216).

No significant sex differences were observed in length of parenteral nutritional length, hospital stay, discharge destination, in-hospital mortality, 6-month mortality or overall mortality by sex (Table 2).

### 3.3. Hydration Status According to Survival

Marked differences in bioimpedance-derived parameters were observed between survivors and non-survivors by sex at 72 h following PN initiation (Table 3).

Among men, non-survivors exhibited significantly lower resistance (428.6 vs. 492.6 Ω, *p* = 0.049) and reactance values (30.7 vs. 41.2 Ω, *p* = 0.004), together with higher hydration percentages (80.9% vs. 76.4%, *p* < 0.001), greater extracellular water proportion (57.3% vs. 52.5%, *p* = 0.015), lower intracellular water proportion (42.7% vs. 47.0%, *p* = 0.028), and higher Na/K ratio (1.75 vs. 1.42, *p* = 0.017).

Similarly, among women, non-survivors demonstrated significantly lower resistance (405.0 vs. 528.2 Ω, *p* = 0.005), reactance (28.2 vs. 41.6 Ω, *p* = 0.005) and phase angle (3.72 vs. 4.48°, *p* = 0.011), accompanied by markedly higher hydration percentages (84.2% vs. 77.2%, *p* < 0.001), greater extracellular water proportion (59.8% vs. 54.5%, *p* = 0.008), lower intracellular water proportion (40.2% vs. 45.5%, *p* = 0.008), and higher Na/K ratio (1.97 vs. 1.39, *p* = 0.011).

Overall, non-survivors exhibited a bioimpedance profile characterized by fluid overload, extracellular fluid expansion and impaired cellular integrity.

**Figure 4:**
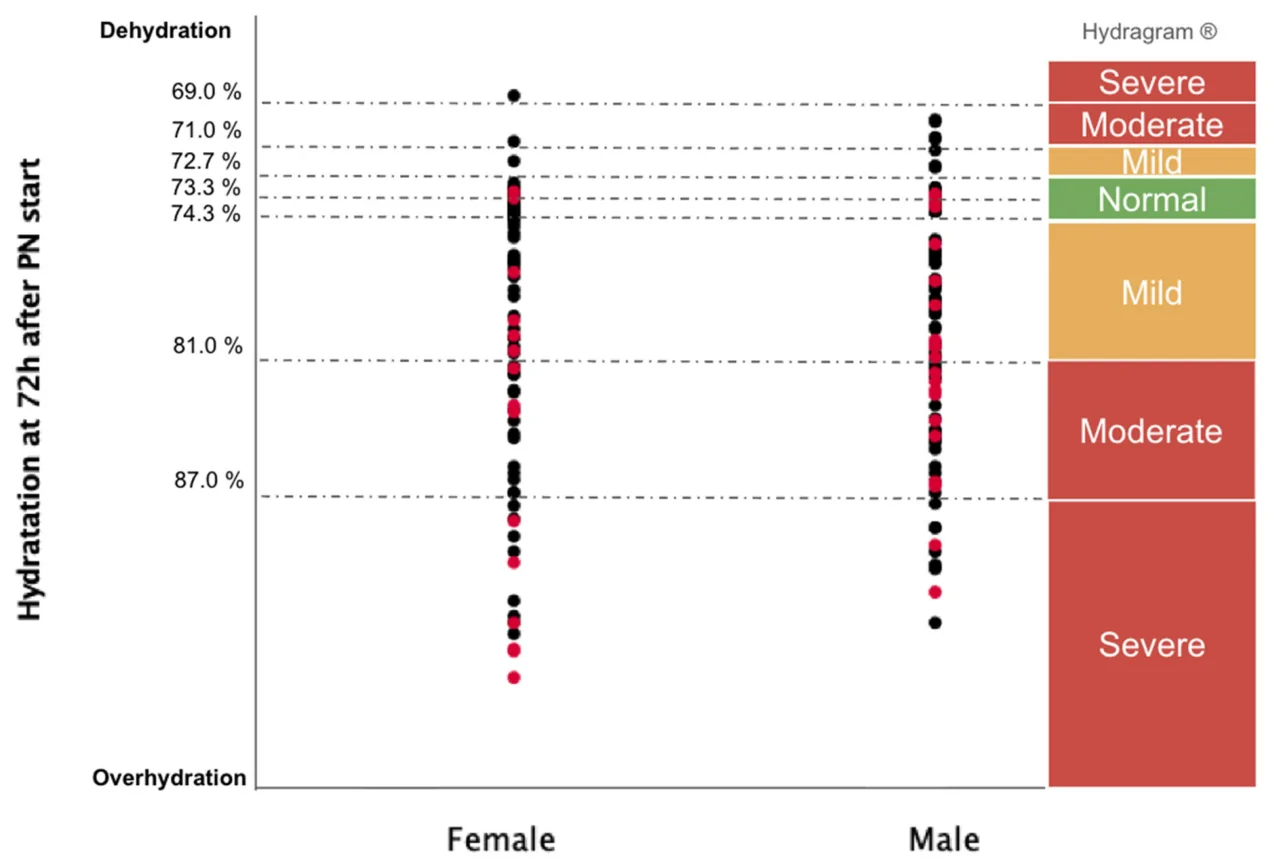
Hydragram values stratified by sex and survival status. Red dots indicate non-survivors and black dots indicate survivors.

To facilitate the clinical interpretation of hydration status, Hydragram^®^ distributions were examined according to survival status (Figure 4). Non-survivors showed a clear displacement toward the hyperhydrated region of the graph, whereas survivors were predominantly distributed within the normohydration range, supporting the association between fluid overload and adverse outcomes.

### 3.4. Diagnostic Performance of Hydration Status

**Figure 5:**
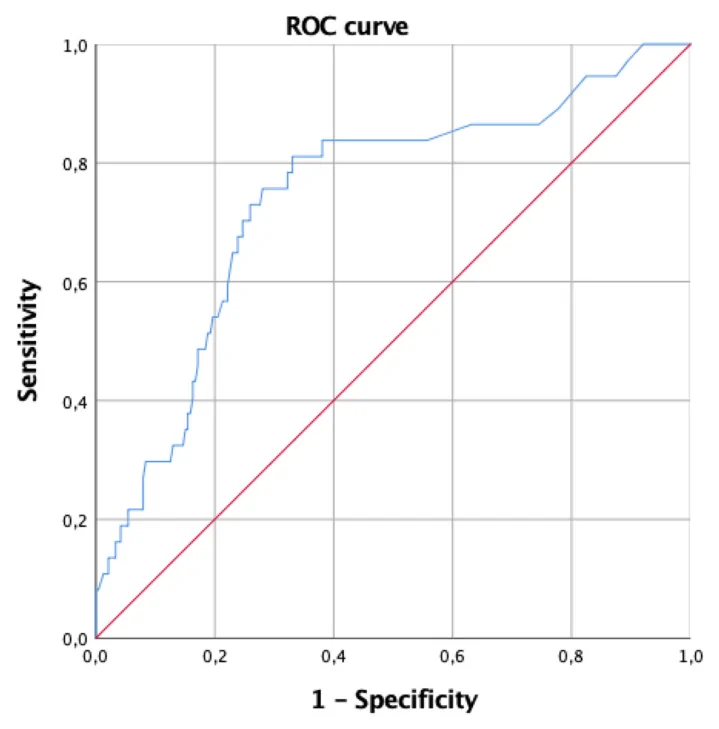
Receiver operating characteristic (ROC) curve of bioimpedance-derived hydration percentage for prediction of in-hospital mortality.

ROC curve analyses demonstrated that hydration percentage measured 72 h after PN initiation was a significant predictor of in-hospital mortality, yielding an AUC of 0.776 (95% CI 0.672–0.880; *p* < 0.001). The optimal cut-off value was 79.45%, providing a sensitivity of 78.3% (Table 4). As illustrated in Figure 5, hydration status showed good discriminative ability for identifying patients at increased risk of in-hospital mortality.

Using the ROC-derived hydration threshold (>79.45%), 34.6% of women (37/107) and 28.4% of men (48/169) were classified as overhydrated, with no significant differences according to sex (*p* = 0.279). In addition, overhydrated patients were less frequently discharged home (31.8% vs. 68.6%, *p* < 0.001).

In contrast, the prevalence of malnutrition according to the GLIM criteria did not differ between groups (41.2% vs. 49.2%, *p* = 0.217). Similarly, no significant differences were observed in hospital length of stay (36.67 ± 32.77 vs. 31.08 ± 30.37 days, *p* = 0.169) or duration of parenteral nutrition (14.68 ± 11.69 vs. 13.64 ± 13.04 days, *p* = 0.529).

### 3.5. Survival Analysis According to Hydration Status

**Figure 6:**
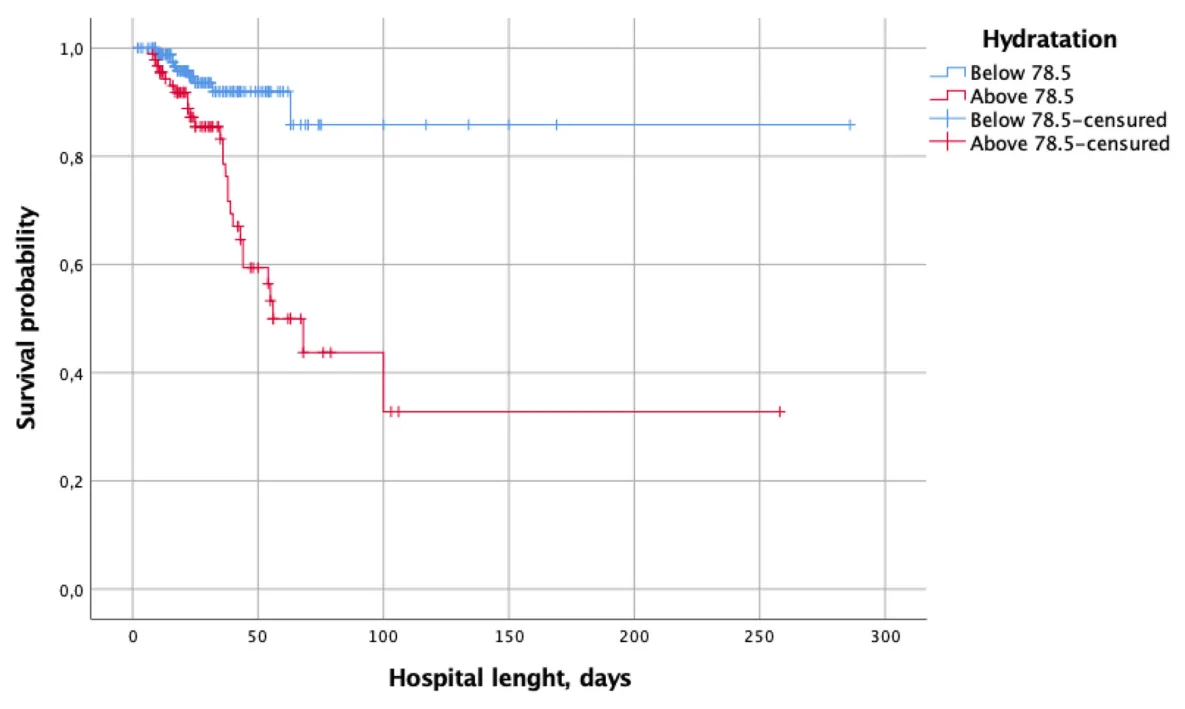
Kaplan–Meier survival curves stratified by hydration status 72 h after initiation of parenteral nutrition.

Kaplan–Meier analysis demonstrated significantly reduced in-hospital survival among overhydrated patients compared with normohydrated patients (log-rank χ^2^ = 18.14, *p* < 0.001; Figure 6). The estimated mean survival time was 117.6 days (95% CI 75.6–159.6) in overhydrated patients versus 251.0 days (95% CI 221.8–280.1) in normohydrated patients.

Median survival was reached only in the overhydrated group and was estimated at 56.0 days (95% CI 39.0–73.0), whereas the median survival time could not be estimated in normohydrated patients due to the low number of events.

Overall, in-hospital mortality occurred in 29.3% of overhydrated patients compared with 5.4% of normohydrated patients.

### 3.6. Independent Predictors of Mortality

Multivariable Cox proportional hazards regression analysis identified age, surgical indication and overhydration as independent predictors of in-hospital mortality (Table 5). Each additional year of age was associated with a 6.4% increase in mortality risk (HR 1.064; 95% CI 1.028–1.102; *p* < 0.001), whereas patients with a surgical indication had a 60.2% lower hazard of death that those without a surgical indication (HR 0.398; 95% CI 0.186–0.854; *p* = 0.018). In contrast, patients classified as overhydrated according to the ROC-derived hydration threshold (>79.45%), exhibited a 2.85-fold higher risk of death compared with normohydrated patients (HR 2.850; 95% CI 1.218–6.669; *p* = 0.016). This association remain significant after adjustment for sex, active oncological disease, duration of fasting prior to PN initiation, inflammatory status, weight loss and skeletal muscle index (SMI-derived from CT body composition assessment).

### 3.7. BIVA Vector Distribution According to Mortality

**Figure 7:**
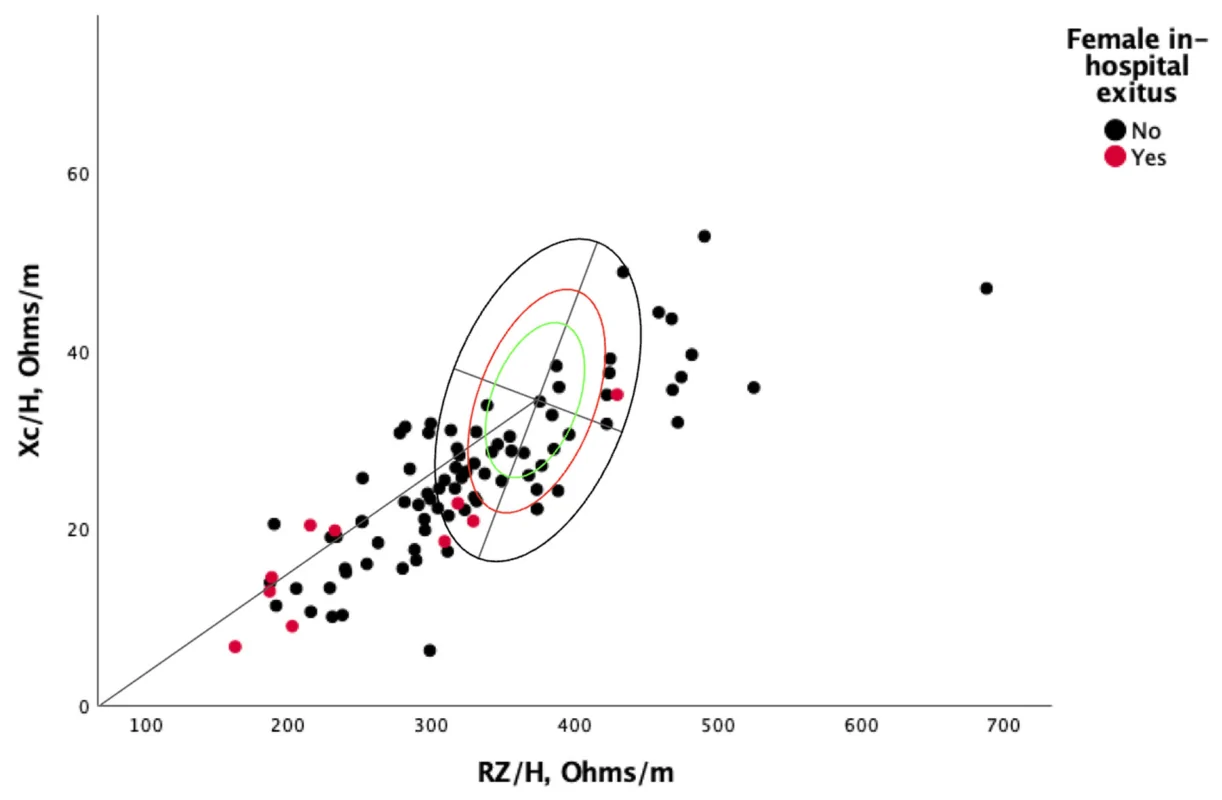
Female-specific bioelectrical impedance vector analysis (BIVA) plot based on the reference tolerance ellipses proposed by Piccoli et al. Ellipses tolerance are represented as 50th green color, 75th red color and 95th black color. Red dots represent non-survivors and black dots represent survivors.

**Figure 8:**
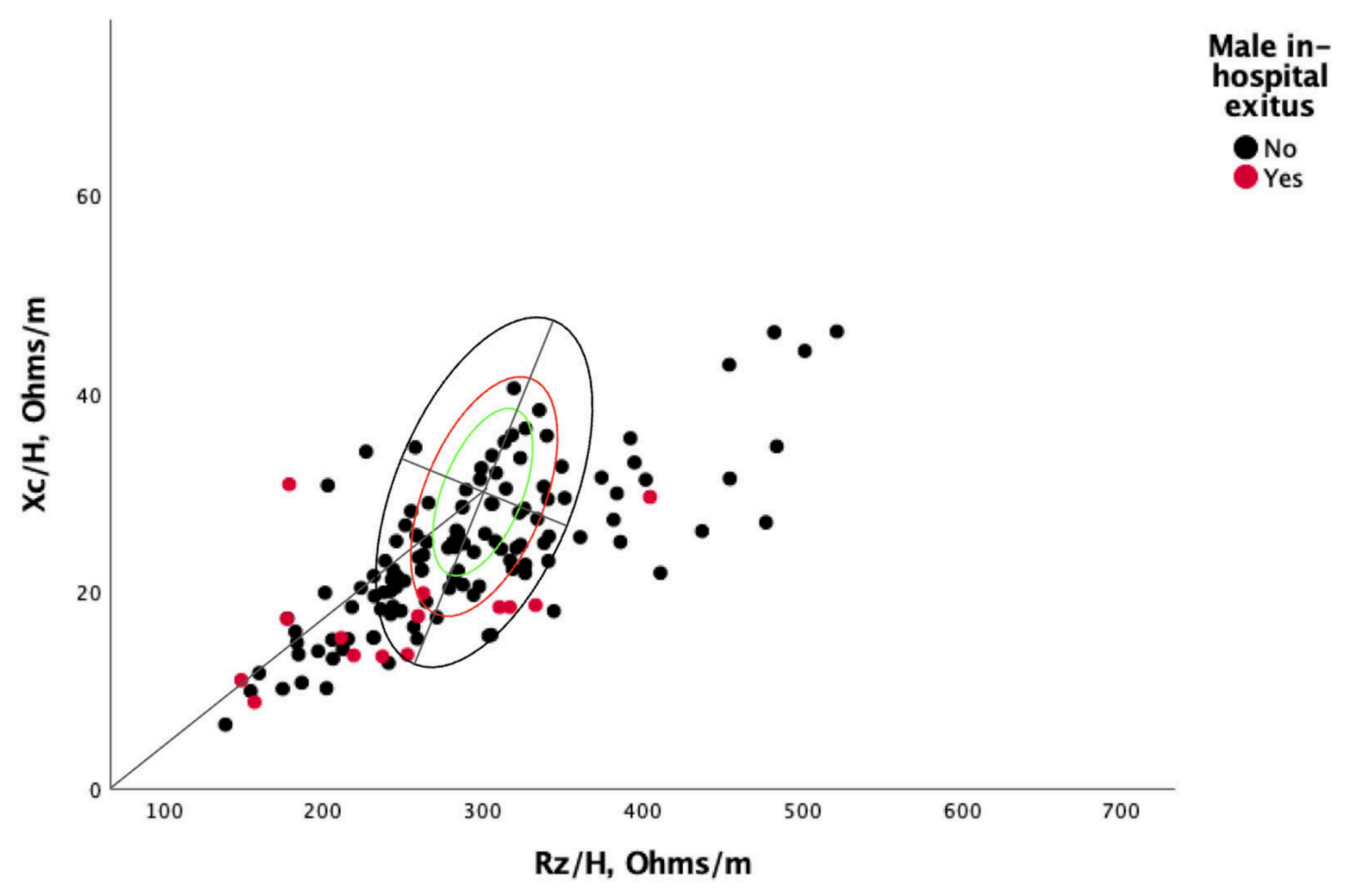
Male-specific bioelectrical impedance vector analysis (BIVA) plot based on the reference tolerance ellipses proposed by Piccoli et al. Ellipses tolerance are represented as 50th green color, 75th red color and 95th black color. Red dots represent non-survivors and black dots represent survivors.

Gender-specific BIVA plots based on Piccoli reference ellipses were generated to visually characterize the relationship between vector position and clinical outcome (Figure 7 for female and Figure 8 for male). In both sexes, non-survivors clustered predominantly in the lower pole of the tolerance ellipses, corresponding to vector positions associated with extracellular fluid expansion and fluid overload. In contrast, survivors were more homogeneously distributed around the central reference area. These findings visually reinforce the association between overhydration and mortality identified by the ROC, showed that non-survivors were predominantly located in the lower region of the tole Kaplan–Meier, and multivariable Cox regression analyses.

## 4. Discussion

The present study identifies bioimpedance-derived overhydration as an independent prognostic marker in hospitalized adults receiving parenteral nutrition. Beyond its association with in-hospital mortality, hydration status remained independently associated with mortality after adjustment for inflammatory burden and CT-derived skeletal muscle index, suggesting that bioimpedance-derived overhydration provides prognostic information beyond the covariates included in our models, although its potential relationship with underlying disease severity cannot be excluded.

Fluid overload has consistently been associated with adverse outcomes in critically ill patients through its effects on tissue perfusion, endothelial dysfunction and organ congestion [35]. However, evidence regarding the prognostic significance of objectively assessed hydration in patients receiving PN remains limited [36,37]. Previous studies have demonstrated similar associations between bioimpedance-derived fluid overload and poor outcomes in intensive care, heart failure and chronic kidney disease [11,22,23,24]. Our findings extend these observations to hospitalized patients receiving PN, a population particularly susceptible to rapid alterations in body water distribution due to surgery, sepsis, gastrointestinal failure and intravenous fluid administration [38]. Furthermore, our results are consistent with the recent cohort reported by Madrid et al., in which morphofunctional assessment identified body composition parameters and handgrip strength as predictors of mortality in hospitalized patients receiving PN [8]. Although hydration was not the primary objective of that study, non-survivors also exhibited significantly greater values of overhydration, reinforcing the hypothesis that extracellular fluid expansion is closely linked to adverse clinical outcomes in this population.

Another noteworthy aspect of our results is that hydration status influences not only prognosis but also the interpretation of body composition. Conventional BIA equations assume a constant hydration of fat-free mass; therefore, extracellular fluid expansion may overestimate lean mass and underestimate the prevalence of low muscle mass. This is consistent with our observation that CT identified a higher prevalence of reduced skeletal muscle than bioimpedance-derived FFMI, highlighting the complementary rather than competing roles of both techniques [20,26,27,31,32]. Whereas CT provides a more robust assessment of muscle quantity in the presence of altered fluid distribution, bioimpedance offers additional information regarding hydration status, which itself appears to be clinically meaningful.

The concordance between Hydragram^®^ and BIVA further strengthens this interpretation. Because BIVA relies on raw bioelectrical measurements rather than predictive equations, the consistent clustering of non-survivors within vectors compatible with extracellular fluid expansion supports the existence of a reproducible bioelectrical phenotype associated with poor prognosis [17,30]. Although updated reference ellipses have recently been proposed [39], the current commercial software continues to use Piccoli’s reference population, making our findings directly applicable to routine clinical practice [30].

Another relevant observation is that the prognostic value of hydration persisted after adjustment for inflammatory status. Acute inflammation profoundly alters both nutritional biomarkers and body water distribution [40,41]; nevertheless, extracellular fluid expansion remained independently associated with mortality, indicating that hydration assessment provides information beyond conventional biochemical markers. These findings support integrating hydration into routine morphofunctional assessment rather than considering it solely as a technical limitation of body composition analysis. Nevertheless, the possibility of residual confounding by underlying disease severity should be acknowledged. In particular, tumor stage was not included in the adjusted models. However, advanced cancer was uncommon in our cohort, with 3.6–5.3% of patients with an oncological diagnosis presenting with a stage beyond IIIb. Furthermore, patients requiring management in specialized units, including those with more severe clinical conditions, were not represented in our study population. Therefore, our findings should be interpreted in the context of the selected population studied, and residual confounding cannot be entirely excluded.

From a clinical perspective, objective hydration assessment may improve early risk stratification in patients receiving PN and facilitate individualized monitoring of fluid and nutritional therapy [42]. However, our observational design does not establish that correction of overhydration improves survival, and prospective interventional studies are required to determine whether bioimpedance-guided fluid management translates into better clinical outcomes.

The main strengths of this study include its prospective design, standardized bioimpedance protocol, complementary CT assessment and multivariable analysis incorporating relevant demographic, clinical, inflammatory, and nutritional covariates. Nevertheless, several limitations should be acknowledged. First, the single-center observational design precludes causal inference, and reverse causality cannot be excluded, as greater underlying disease severity may contribute to fluid accumulation and adverse outcomes. Residual confounding, including by tumor stage, also remains possible. Second, fluid intake and output data were not systematically collected, preventing us from quantifying fluid balance and determining the extent to which preceding intravenous fluid administration may have influenced bioimpedance-derived hydration estimates. Third, patients requiring management in specialized units were not recruited, limiting the generalizability of our findings to more severely ill patients. Finally, the proposed hydration cut-off requires external validation, as its prognostic performance was assessed in the present cohort and potential optimism cannot be excluded. Therefore, our findings primarily support the prognostic relevance of hydration status in relation to in-hospital mortality, whereas its value for predicting longer-term outcomes remains to be established. Multicenter prospective studies including a broader spectrum of clinical severity are warranted to validate these findings and clarify the independent prognostic value and clinical applicability of bioimpedance-derived overhydration.

## 5. Conclusions

Bioimpedance-derived overhydration is an independent predictor of in-hospital mortality in patients receiving parenteral nutrition. Beyond its influence on body composition estimates, hydration should be considered a clinically meaningful component of morphofunctional assessment, providing complementary prognostic information that may improve early risk stratification in this high-risk population.

## Figures and Tables

**Table 1:** General features of the sample by sex.

		Men (*n* = 169)	Women (*n* = 107)	*p* Value
Baseline characteristics				
Age (years)	m ± SD	65.58 ± 16.8	66.01 ± 17.75	0.840
Oncological patient (*n* = 150)	*n* (%)	94 (55.6)	56 (52.3)	0.593
Cancer Diagnosis	*n* (%)			0.280 *
Colorectal Cancer		41 (43.6)	14 (25.0)	
Pancreatic Cancer		6 (6.4)	5 (8.9)	
Gastric Cancer		7 (7.4)	7 (12.5)	
Urologic Malignancies		14 (14.9)	4 (7.1)	
Esophageal Cancer		10 (10.6)	3 (5.5)	
Myeloid Leukemia		11 (11.8)	12 (21.4)	
Ovarian Cancer		0 (0.0)	7 (12.5)	
Lung Cancer		4 (4.2)	4 (7.1)	
Osteosarcoma		1 (1.1)	0 (0.0)	
Disease stage (>IIIb)	*n* (%)	5 (5.3)	2 (3.6)	0.436
Surgical indication (*n* = 154)	*n* (%)	97 (57.4)	57 (53.3)	0.501
Days without oral intake	m ± SD	7.06 ± 5.01	6.68 ± 4.79	0.537
GLIM criteria	*n* (%)	79 (46.7)	50 (46.7)	0.998
BMI (Kg/m^2^)	m ± SD	24.56 ± 4.69	25.57 ± 6.12	0.149
Low BMI	*n* (%)	38 (22.5)	20 (18.7)	0.451
Fat-Free Mass Index (Kg/m^2^)	m ± SD	18.81 ± 2.63	17.14 ± 2.52	**<0.001**
Low FFMI	*n* (%)	40 (23.7)	12 (11.2)	**0.010**
Loss Weight (%)	m ± SD	4.03 ± 6.97	5.62 ± 9.43	0.137
Refeeding risk	*n* (%)			
Minor Risk A		131 (77.5)	83 (77.6)	0.991
High Risk B		92 (54.4)	64 (59.8)	0.380
Very High Risk C		23 (13.6)	17 (15.9)	0.600
Biochemical parameters	m ± SD			
Albumin, g/dL		3.20 ± 2.13	3.05 ± 0.56	0.475
Prealbumin, mg/dL		10.83 ± 6.44	9.88 ± 5.16	0.184
CRP, mg/dL		13.21 ± 9.82	12.50 ± 10.81	0.577
CRP/prealbumin ratio		2.02 ± 2.12	2.01 ± 2.35	0.972
PO4, mg/dL		2.90 [1.3]	2.70 [1.3]	0.786
PN indication	*n* (%)			0.324 *
Gastrointestinal obstruction or dysmotility		73 (43.2)	41 (38.3)	
Feeding intolerance or inability to swallow		48 (28.4)	26 (24.3)	
Severe mucositis		8 (4.7)	10 (9.3)	
Gastrointestinal bleeding		6 (3.6)	3 (2.9)	
Chronic severe diarrhea		3 (1.8)	1 (0.9)	
Bowel rest		30 (17.8)	25 (23.4)	
Severe acute pancreatitis		1 (0.5)	1 (0.9)	
Initial PN Energy, kcal/kg	m ± SD	15.72 ± 3.91	17.43 ± 4.04	**0.001**
CT-derived body composition (*n* = 256)				
SMI	m ± SD	49.79 ± 10.56	46.07 ± 8.33	**0.003**
Low SMI, [34]	*n* (%)	68 (44.2)	25 (25.3)	**0.002**
SMD	m ± SD	21.31 ± 17.64	16.69 ± 18.08	**0.045**
Low muscle radiodensity, [34]	*n* (%)	122 (79.2)	83 (83.8)	0.361
SATI	m ± SD	45.41 ± 22.96	87.28 ± 47.81	**<0.001**
SATD	m ± SD	−78.99 ± 19.22	−73.72 ± 17.49	0.757
VATI	m ± SD	62.54 ± 40.77	47.06 ± 38.87	**0.003**
VATD	m ± SD	−78.99 ± 19.22	−73.72 ± 17.49	**0.028**
IMATI	m ± SD	4.32 ± 3.90	5.20 ± 4.88	0.116
IMATD	m ± SD	−69.10 ± 7.78	−74.45 ± 63.80	0.305

Abbreviations: BMI = Body Mass Index; m = mean; FFMI = Fat-Free Mass Index; CT = Computed Tomography; SMI = Skeletal Muscle Index; SMD = Skeletal Muscle Radiodensity; SATI = Subcutaneous Adipose Tissue Index; SATD = Subcutaneous Adipose Tissue Radiodensity; VATI = Visceral Adipose Tissue Index; VATD = Visceral Adipose Tissue Radiodensity; IMATI = InterMuscular Adipose Tissue Index; IMATD = InterMuscular Adipose Tissue Radiodensity; GLIM = Global Leadership Initiative on Malnutrition; CRP = C-Reactive Protein; PO4 = phosphate; PN = Parenteral Nutrition; SD = Standard Deviation; Bold values indicate a significant *p*-value < 0.05. * Bonferroni correction was applied and no significant differences were found between groups.

**Table 2:** Biochemical parameters, Bioimpedance-derived parameters 72 h hours after parenteral nutrition treatment started, and Clinical Outcomes by sex.

		Men (*n* = 169)	Women (*n* = 107)	*p* Value
Biochemical parameters				
PO4 72 h, mg/dL	Median (IR)	2.70 [1.2]	2.6 (1.4)	0.124
PO4 reduction at 72 h, %	Median (IR)	7.01 [44.7]	7.14 (49)	0.085
Bioimpedance 72 h after PN	m ± SD			
Rz, ohms		486.05 ± 121.37	514.91 ± 132.54	0.090
Xc, ohms		40.16 ± 13.20	40.13 ± 14.37	0.988
PA, º		4.72 ± 1.16	4.40 ± 0.91	**0.026**
Hydration, %		77.01 ± 5.19	78.20 ± 6.47	0.093
TBW, %		61.17 ± 8.94	55.53 ± 12.69	**<0.001**
ECW, %		53.03 ± 6.93	55.03 ± 6.11	**0.025**
ICW,%		46.57 ± 6.92	44.96 ± 6.10	0.070
BCM, kg		25.80 ± 7.31	19.58 ± 4.31	**<0.001**
Na/K		1.45 ± 0.49	1.45 ± 0.68	0.997
BMR, kcal per day		1500.02 ± 212.45	1305.05 ± 175.46	**<0.001**
Clinical outcomes				
Refeeding	*n* (%)	47 (27.8)	42 (39.3)	**0.048**
PN length, days	m ± SD	14.01 ± 13.28	13.89 ± 11.57	0.937
Hospital length, days	m ± SD	34.79 ± 35.47	29.66 ± 22.61	0.184
Discharge home	*n* (%)	101 (59.8)	57 (53.3)	0.633
Intrahospital exitus	*n* (%)	22 (13.0)	15 (14.0)	0.812
6 months follow-up exitus	*n* (%)	19 (11.2)	11 (10.3)	0.802
Overall mortality	*n* (%)	41 (24.2)	26 (24.3)	0.905

Abbreviations: BMI = Body Mass Index; m = mean; SD = Standard Deviation; Rz = Resistance; Xc = Reactance; PA = Phase Angle; TBW = Total Body Water; ECW = Extra Cellular Water; ICW = Intra Cellular Water; BCM = Body Cell Mass; Na/K = Sodium/Potassium ratio; PN = Parenteral Nutrition. Bold values indicate a significant *p*-value < 0.05.

**Table 3:** Bioimpedance-derived parameters 72 h after parenteral nutrition treatment started according to vital status by sex.

	Men (*n* = 169)			Women (*n* = 107)		
	Survival (*n* = 147)	Non-Survival (*n* = 22)	*p* Value	Survival (*n* = 92)	Non-Survival (*n* = 15)	*p* Value
Rz, ohms	492.59 ± 119.26	428.58 ± 129.03	**0.049**	528.15 ± 126.81	405.04 ± 134.21	**0.005**
Xc, ohms	41.23 ± 12.94	30.69 ± 12.08	**0.004**	41.56 ± 14.00	28.23 ± 12.29	**0.005**
PA, º	4.78 ± 1.07	4.20 ± 1.73	**0.061**	4.48 ± 0.88	3.72 ± 0.86	**0.011**
Hydration, %	76.43 ± 4.49	80.89 ± 5.22	**<0.001**	77.23 ± 5.74	84.20 ± 7.64	**<0.001**
TBW, %	60.85 ± 8.93	63.97 ± 8.87	0.218	55.09 ± 11.79	59.19 ± 19.07	0.338
ECW, %	52.54 ± 6.61	57.26 ± 8.41	**0.015**	54.46 ± 5.78	59.83 ± 6.91	**0.008**
ICW,%	47.01 ± 6.64	42.73 ± 8.37	**0.028**	45.53 ± 5.79	40.16 ± 6.85	**0.008**
BCM, kg	25.76 ± 6.38	26.19 ± 13.31	0.835	19.78 ± 4.29	17.90 ± 4.32	0.193
Na/K	1.42 ± 0.48	1.75 ± 0.47	**0.017**	1.39 ± 0.53	1.97 ± 1.34	**0.011**

Abbreviations: Rz = Resistance; Xc = Reactance; PA = Phase Angle; TBW = Total Body Water; ECW = Extra Cellular Water; ICW = Intra Cellular Water; BCM = Body Cell Mass; Na/K = Sodium/Potassium ratio. Results are expressed as m ± SD. Bold values indicate a significant *p*-value < 0.05.

**Table 4:** Optimal probability cut-off point for in-hospital mortality.

Variable	Cut-Off Point	Sensitivity	1 − Specificity	AUC	95% CI for OR	*p* Value
Hydration after PN (72 h)	79.45%	0.783	0.263	0.776	0.672–0.880	**<0.001**

Abbreviations: PN = Parenteral Nutrition; AUC: Area Under Curve; OR: Odd Ratio. Bold values indicate a significant *p*-value < 0.05.

**Table 5:** Multivariable Cox proportional hazards regression analysis for in-hospital mortality.

Variable	HR (95% CI)	*p* Value
Age	1.064 (1.028–1.102)	**<0.001**
Sex (1 = male)	1.104 (0.529–2.303)	0.793
Surgical indication (1 = yes)	0.398 (0.186–0.854)	**0.018**
Oncological patient (1 = yes)	0.949 (0.465–1.939)	0.793
Days without oral intake	1.011 (0.940–1.088)	0.176
Weight loss, %	0.967 (0.914–1.022)	0.769
Overhydration (>79.45%) (1 = yes)	2.852 (1.213–6.704)	**0.016**
CRP/prealbumin ratio	1.157 (0.999–1.342)	0.052
Skeletal Muscle Index	0.980 (0.939–1.023)	0.350

Abbreviations: HR = Hazard Ratio; CRP = C-Reactive Protein; Data are presented as HR with 95% confidence intervals (95% CI). Overhydration was defined according to the Receiver operating characteristic (ROC)-derived hydration threshold (>79.45%). Overall model χ^2^ = 29.55, *p* < 0.001. Bold values indicate a significant *p*-value < 0.05.

## Data Availability

All data were pseudonymized using secure protocols to ensure patient confidentiality. Due to the sensitive nature of the clinical information and institutional agreements, the dataset is not publicly available. However, identified data may be made available from the corresponding author upon reasonable request and with permission from the participating centers and relevant ethics committees.

## Abbreviations

The following abbreviations are used in this manuscript:

BC	Body Composition
BMI	Body Mass Index
CT	Computed Tomography
GLIM	Global Leadership Initiative on Malnutrition
LMMA	Lean Muscle Mass Area
LMMI	Lean Muscle Mass Index
LMD	Lean Muscle Radiodensity
SMI	Skeletal Muscle Index
SMA	Skeletal Muscle Area
SMD	Skeletal Muscle Radiodensity
SAT	Subcutaneous Adipose Tissue
SATI	Subcutaneous Adipose Tissue Index
SATD	Subcutaneous Adipose Tissue Radiodensity
VAT	Visceral Adipose Tissue
VATI	Visceral Adipose Tissue Index
VATD	Visceral Adipose Tissue Radiodensity
IMAT	Intermuscular Adipose Tissue
IMATI	Intermuscular Adipose Tissue Index
IMATD	Intermuscular Adipose Tissue Radiodensity
FFMI	Fat-Free Mass Index
BIA	Bioelectrical Impedance
IR	Interquartile Range
SD	Standard Deviation
